# MALDI-TOF-MS Analysis in the Identification of Urine Proteomic Patterns of Gestational Trophoblastic Disease

**DOI:** 10.3390/metabo9020030

**Published:** 2019-02-09

**Authors:** Paulina Banach, Paweł Dereziński, Eliza Matuszewska, Jan Matysiak, Hubert Bochyński, Zenon J. Kokot, Ewa Nowak-Markwitz

**Affiliations:** 1 Gynecologic Oncology Department, Poznan University of Medical Sciences, Polna 33, 60-535 Poznan, Poland; h.bochynski@gmail.com (H.B.); ewamarkwitz@poczta.fm (E.N.-M.); 2 Department of Inorganic and Analytical Chemistry, Poznan University of Medical Sciences, Grunwaldzka 6, 60-780 Poznan, Poland; p.derezinski@gmail.com (P.D.); eliza.matuszewska@gmail.com (E.M.); jmatysiak@ump.edu.pl (J.M.); zkokot@ump.edu.pl (Z.J.K.)

**Keywords:** gestational trophoblastic disease, biomarkers, MALDI-TOF-MS, protein–peptide profiling

## Abstract

Gestational trophoblastic disease (GTD) is a group of highly aggressive, rare tumors. Human chorionic gonadotropin is a common biomarker used in the diagnosis and monitoring of GTD. To improve our knowledge of the pathology of GTD, we performed protein-peptide profiling on the urine of patients affected with gestational trophoblastic neoplasm (GTN). We analyzed urine samples from patients diagnosed with GTN (*n* = 26) and from healthy pregnant and non-pregnant controls (*n* = 17) using matrix-assisted laser desorption/ionization time-of-flight mass spectrometry (MALDI-TOF-MS). Ions were examined in a linear mode over a *m*/*z* range of 1000–10,000. All GTN urine samples were analyzed before and after treatment and compared with those of the controls. The statistical analyses included multivariate classification algorithms as well as ROC curves. Urine sample analyses revealed there were significant differences in the composition of the ions between the evaluated groups. Comparing the pre-treatment and group with the pregnant controls, we identified two discriminatory proteins: hemoglobin subunit α (*m*/*z* = 1951.81) and complement C4A (*m*/*z* = 1895.43). Then, comparing urine samples from the post-treatment cases with those from the non-pregnant controls, we identified the peptides uromodulin fragments (*m*/*z* = 1682.34 and 1913.54) and complement C4A (*m*/*z* = 1895.43).

## 1. Introduction

Proteomics is an analytical tool used for protein biomarker characterization and clinical diagnostics. Protein biomarkers, such as human chorionic gonadotropin β (βhCG), carbohydrate antigen 125 (CA 125), and human epididymis protein 4 (HE4), are already used in the field of gynecologic oncology [1]. Proteomics is a promising method for discovering protein profiles and increasing the sensitivity and specificity of biomarkers that are currently in use [2].

Over the years, it has been an ongoing challenge to find biomarkers of gestational trophoblastic disease (GTD) that could be used for better identification of patients who are at risk of the disease becoming more aggressive, and for the earlier administration of proper treatment. GTD is a heterogeneous group of benign and malignant pathologies that arise from trophoblastic cells. Hydatidiform mole (HM) is a benign form of the disease where the only treatment choice is tissue evacuation from the uterine cavity. About 20% of HM may transform into the malignant form, therefore requiring further chemotherapy [3]. Factors involved in the transformation of HM into neoplastic process, known as gestational trophoblastic neoplasm (GTN), remain poorly understood. GTN includes invasive mole, choriocarcinoma, placental site trophoblastic tumor (PSTT), and epithelial trophoblastic tumor (ETT). Malignant gestational trophoblastic disease misdiagnosed at an early stage may metastasize to distant tissues. Treatment of GTN depends primarily on an early diagnosis and an applicable treatment regimen. WHO proposed a Prognostic Scoring System for the identification of patients who are at high risk of developing aggressive GTN. Accordingly, GTN is divided into two groups: low-risk and high-risk disease. This scoring system includes clinical features such as age, time since last pregnancy, pretreatment beta subunit of human chorionic gonadotropin (βhCG) level, form of preceding pregnancy, tumor size, site of spread, number of metastases, and number of drugs used so far that failed to treat the tumor. Low-risk patients may be managed with methotrexate therapy, but those scored as high-risk disease require multi-agent chemotherapy [4].

Proteomics patterns in gynecological oncology have been investigated in many malignancies [5,6,7,8], including GTD [9,10,11]. To the best of our knowledge, we are the first to analyze protein profiles by MALDI-TOF-MS in the urine of patients suffering from GTN. Identification of the disease markers in urine is a potentially non-invasive method for establishing diagnosis and monitoring treatment. The aim of this study was to establish whether peptide profiles were altered in gestational trophoblastic disease in comparison with controls.

## 2. Methods

### 2.1. Patient Characteristics

Urine samples were collected between July 2014 and December 2016 in the Department of Gynecology, Obstetrics, and Gynecologic Oncology of Poznan University of Medical Sciences, which is the Polish Reference Center of GTD. A study group of 26 patients diagnosed with GTN was compared with a group of 17 healthy controls. All the women enrolled in the study were non-smokers, had a BMI within the norm (18.5–24.99), and were of reproductive age (21–35 years). The control subjects were divided into 2 subgroups: One of 9 pregnant patients and the other of 8 non-pregnant patients. The pregnant women of the control group were enrolled during the first trimester of their pregnancy (the 9th and 10th weeks of gestational age). The non-pregnant control group was comprised of final-year Medical Studies students (25–26 years old). Patients with GTN were treated with one of the following alone: methotrexate, actinomycin, or EMA-Co regimen (etoposide, methotrexate, actinomycin, cyclophosphamide, and vincristine). Urine samples were collected prior to treatment and after the completion of chemotherapy. In our study, a first morning sample for all individuals included in the study was collected. All the participants were instructed to provide a “mid-stream” sample. After collection, samples were immediately stored at −80 °C. All the samples were stored in identical vials until analysis. The samples from patients and students (controls) were collected at the hospital. For those women after GTN treatment, samples were provided three weeks after their last chemotherapy.

The study was conducted in accordance with the Declaration of Helsinki and the protocol was approved by the Ethical Review Board of Poznan University of Medical Sciences, Poland (Decision No. 425/14). A written consent for inclusion was obtained from all participants prior to sample collection.

### 2.2. Mass Spectrometry Analysis

Mass spectrometry (MS) analysis was performed using the nanoLC-MALDI-TOF/TOF-MS/MS method, which generated protein-peptide profiles characteristic of each of the study groups and enabled the identification of discriminating peaks. Urine samples were normalized using urine-specific gravity prior to MS analysis. Urine-specific gravity, which is the ratio of the density of a urine sample to the density of water, was measured precisely using a hand-held urine refractometer (ATAGO, Tokyo, Japan). Urine samples were subsequently diluted with water to the lowest measured specific gravity (1.003) in the population of samples prior to the protein-peptide profiling.

MS analyses were preceded by purification and concentration of the urine samples. Pretreatment of the samples was performed using ZipTip C18 (Millipore, Bedford, MA, USA) reversed-phase chromatography micropipette tips. 9 µL of each urine sample (normalized using urine-specific gravity) was mixed with 1 µL of 0.1% trifluoroacetic acid (TFA). Then, the mixtures were loaded onto ZipTip tips. For conditioning the tips, acetonitrile (ACN) and 0.1% TFA were used. After washing with 0.1% TFA, bound peptides were eluted with 50% ACN in 0.1% TFA. For the MALDI-TOF-MS protein-peptide profiling, 1 µL of each of the pretreated samples was manually spotted onto the AnchorChip Standard Plate (Bruker Daltonics, Bremen, Germany) in triplicates. Then, each spot was covered with 1 µL of the matrix solution (0.7 g/L solution of alpha-cyano-4-hydroxycinnamic acid (HCCA) in a mixture containing 85% of ACN, 15% of water, 0.1% of TFA and 1 mM of ammonium phosphate). MS analysis was performed using the UltrafleXtreme (Bruker Daltonics, Bremen, Germany) mass spectrometer working in a linear mode. Ions were analyzed in the range of *m*/*z* 1000–10,000. One spectrum was acquired for each spot by accumulating 2000 laser shots per spectrum. External calibration was performed using a standard mixture of Protein Calibration Standard I and Peptide Calibration Standard (Bruker) (5:1, *v/v*). The average deviation from the reference masses was not greater than 100 ppm. The recorded spectra were subsequently analyzed using ClinProTools 3.0 software (Bruker). Data processing included the Benjamini and Hochberg p-value adjustment procedure to deal with the multiple hypothesis testing problems. Our statistical analyses included multivariate classification algorithms (genetic algorithm, supervised neural network, and quick classifier) and ROC curves. The analyses allowed us to select several peptide candidates for subsequent identification.

For the identification of the differentiating peptides, the nanoLC-MALDI-TOF/TOF MS/MS system was used. First, urine samples were pretreated with ZipTip C18 pipette tips and then subjected to nanoLC separation. The nanoLC set parts were: EASY-nLC II (Bruker), nanoflow HPLC system, and Proteineer-fc II (Bruker) collector of fractions. The nanoLC system consisted of a NS-MP-10 BioSphere C18 (NanoSeparations) trap column (20 mm × 100 µm I.D., particle size 5 µm, pore size 120 Å), and an Acclaim PepMap 100 (Thermo Scientific) column (150 mm × 75 µm I.D., particle size 3 µm, pore size 100 Å). The gradient elution method was: 2%–50% of ACN in 96 min (mobile phase A—0.05% TFA water solution and mobile phase B—0.05% TFA in 90% ACN). 4 µL of the sample was injected into the column and the separation flow rate was 300 nL/min. The nanoLC separation resulted in 384 fractions. Each of the obtained fractions was mixed with a matrix solution containing 36 µL of HCCA saturated solution in 0.1% TFA and acetonitrile (90:10 *v*/*v*), 748 µL of acetonitrile and 0.1% TFA (95:5 *v*/*v*) mixture, 8 µL of 10% TFA, and 8 µL of 100 mM ammonium phosphate. The sample-and-matrix mixture was automatically spotted onto AnchorChip Standard Plate by the fraction collector. MS analysis was performed using an UltrafleXtreme (Bruker) mass spectrometer working in a reflector mode in the range of *m*/*z* 700–3500. Peptide Calibration Standard (Bruker) mixture was used for the calibration. For the acquisition of spectra, FlexControl 3.4 (Bruker) software was used. The data obtained was processed with FlexAnalysis 3.4 (Bruker). The protein database search was performed using BioTools 3.2 (Bruker). For the protein identification, a SwissProt database and Mascot 2.4.1 search engine with taxonomic restriction to *Homo sapiens* were applied. The protein search parameters were: fragment ion mass tolerance *m*/*z* ±0.7, precursor ion mass tolerance ±50 ppm, mono isotopic mass, and peptide charge +1. Automated de novo sequencing combined with database searching was performed with precursor and fragment mass error tolerances of *m*/*z* 0.7. The following modifications were considered: Carbamidomethylation of cysteine as a fixed modification and oxidation of methionine as a variable modification.

## 3. Results

**Figure 1:**
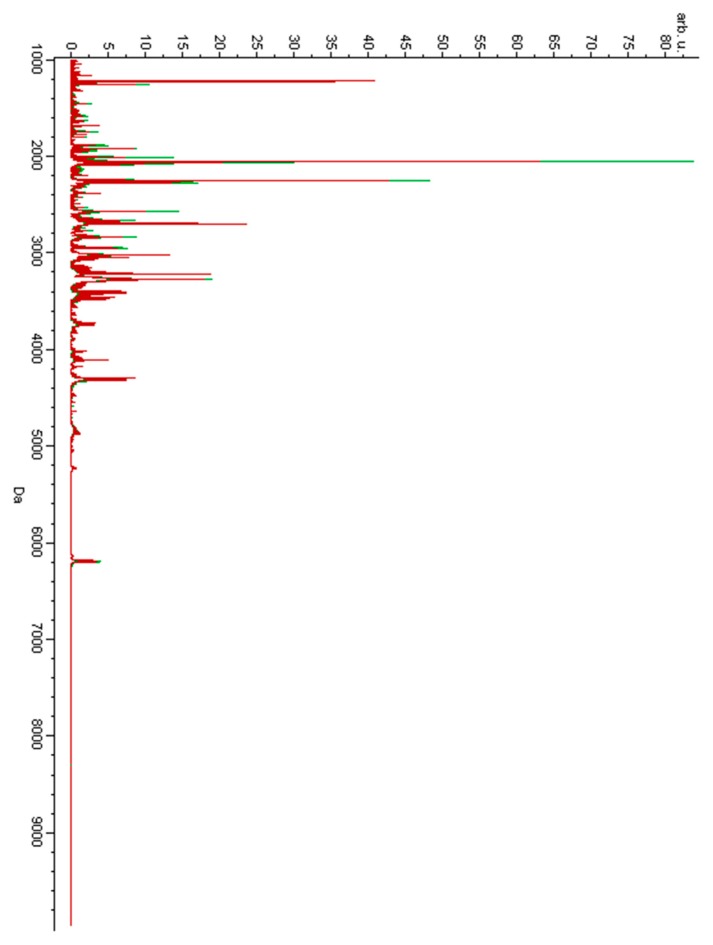
Average MALDI-TOF mass spectra of urine samples of the women suffering from gestational trophoblastic neoplasm (GTN) before treatment (red) and of the pregnant controls (green) over the full scan range of *m*/*z* 1000–10,000.

**Figure 2:**
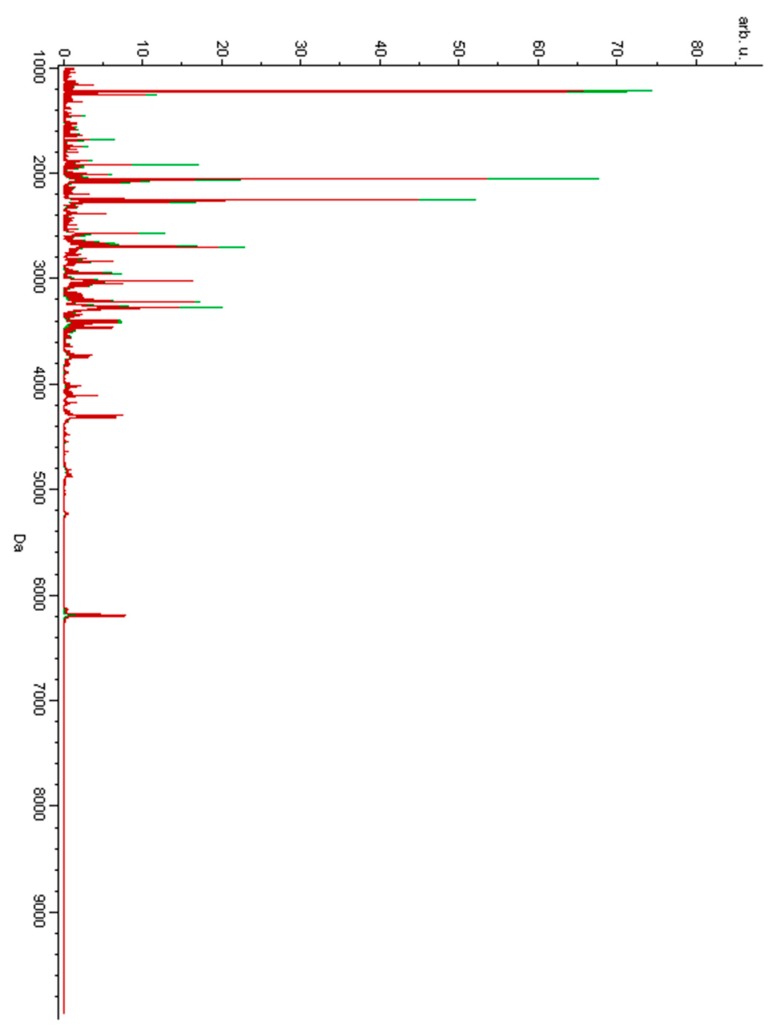
Average MALDI-TOF mass spectra of urine samples of the women suffering from GTN after treatment (red) and of the non-pregnant controls (green) over the full scan range of *m*/*z* 1000–10,000.

Urine samples were analyzed using the MALDI-TOF-MS technique. Figure 1 presents the overlaid average MALDI-TOF mass spectra characteristic of urine samples of the women diagnosed with GTN before treatment and of the pregnant controls. Figure 2 presents the overlaid average spectra of urine samples of the women after GTN treatment and of the non-pregnant controls.

Analysis of urine samples revealed differences in the protein/peptide composition when comparing the study groups. To distinguish the groups, three different algorithms were applied: genetic algorithm (GA), supervised neural network (SNN), and quick classifier (QC). The genetic algorithm model was found to be the most discriminative. For this algorithm, we calculated the cross validation and recognition capability (measuring the reliability of the model).

**Figure 3:**
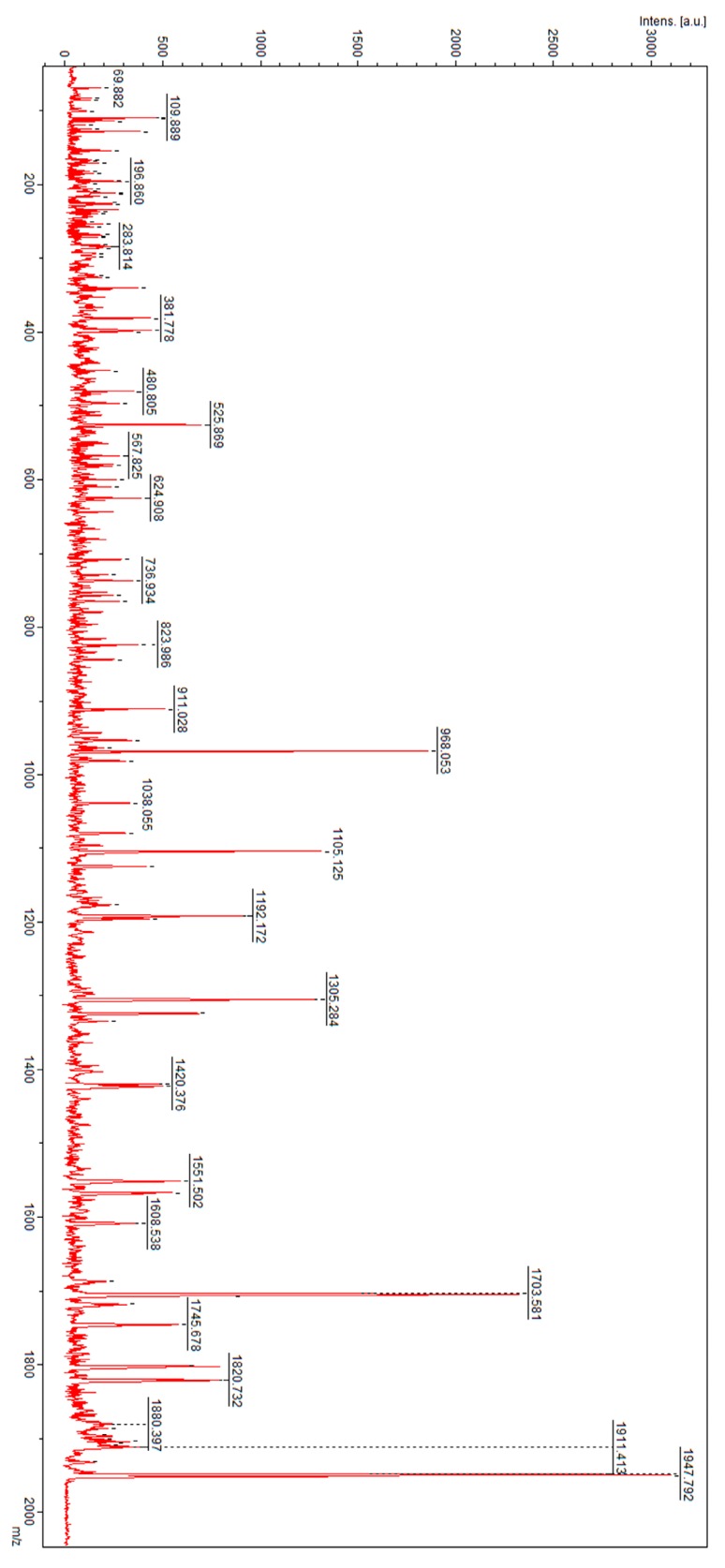
Fragmentation spectrum of precursor ion of *m*/*z* 1951.81 identified as hemoglobin subunit α.

**Figure 4:**
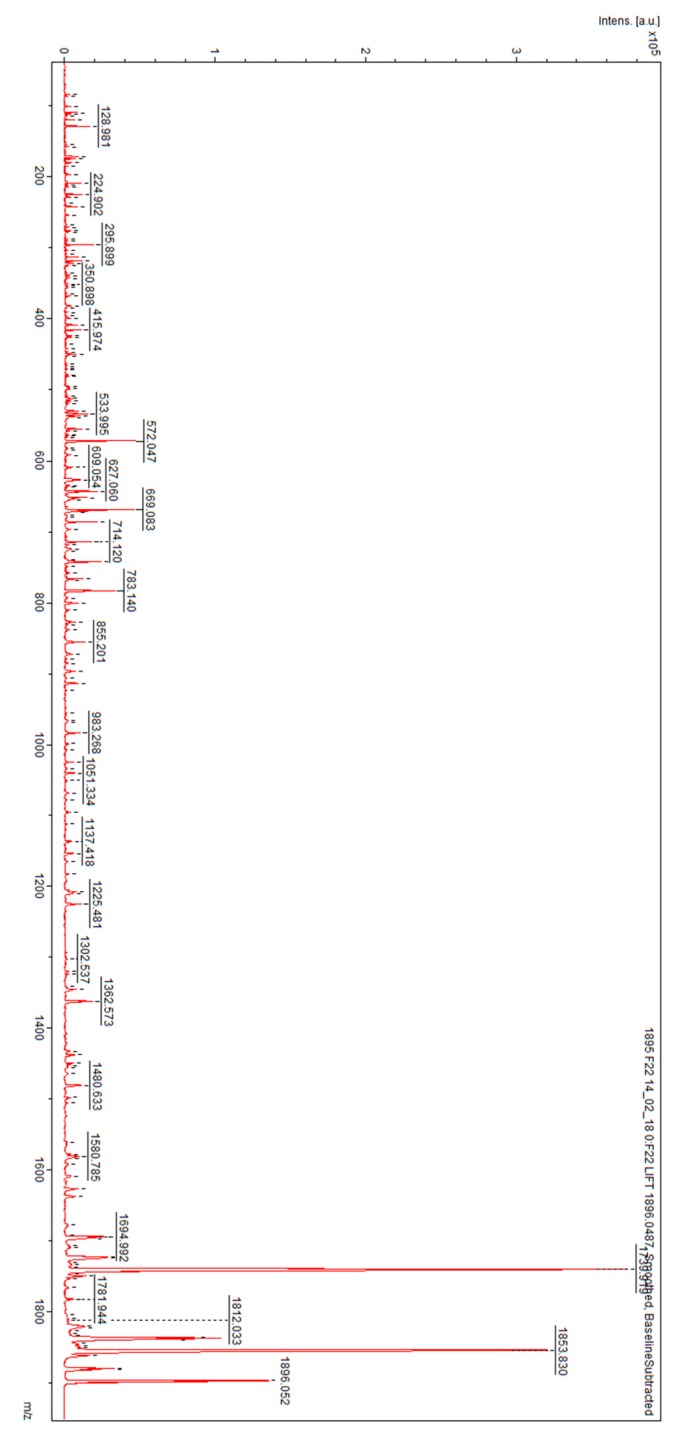
Fragmentation spectrum of precursor ion of *m*/*z* 1895.43 identified as complement C4A.

When comparing sample results from the women suffering from GTN prior to treatment and those from the pregnant controls, the average value of cross validation obtained from three repetitions was 68.2% and the recognition capability was 94.4% (Table 1). Peaks of *m*/*z* 1162.66, 1270.44, 1525.03, 1895.25, 1936.19, 1951.81, 2010.43, 2033.35, 2698.14, 3015.25, 3154.99, 3388.61, 4866.94, 8600.7, and 9148.9 were classified as discriminatory. These *m*/*z* features were subjected to direct nanoLC-MALDI-TOF/TOF MS/MS analysis for identification. The analysis resulted in fragmentation spectra which were then analyzed with a SwissProt peptide sequence database and a Mascot search engine. The analysis of *m*/*z* 1951.81 resulted in identification of hemoglobin subunit α (HBA_HUMAN) with the peptide sequence of Y.FPHFDLSHGSAQVKGHGK.K. Complement C4A (CO4A_HUMAN) was identified for a peak of *m*/*z* 1895.43 with the following sentence: R.NGFKSHALQLNNRQIR.G. The fragmentation spectra of *m*/*z* 1951.81 and 1895.43 are presented in Figure 3 and Figure 4, respectively.

**Figure 5:**
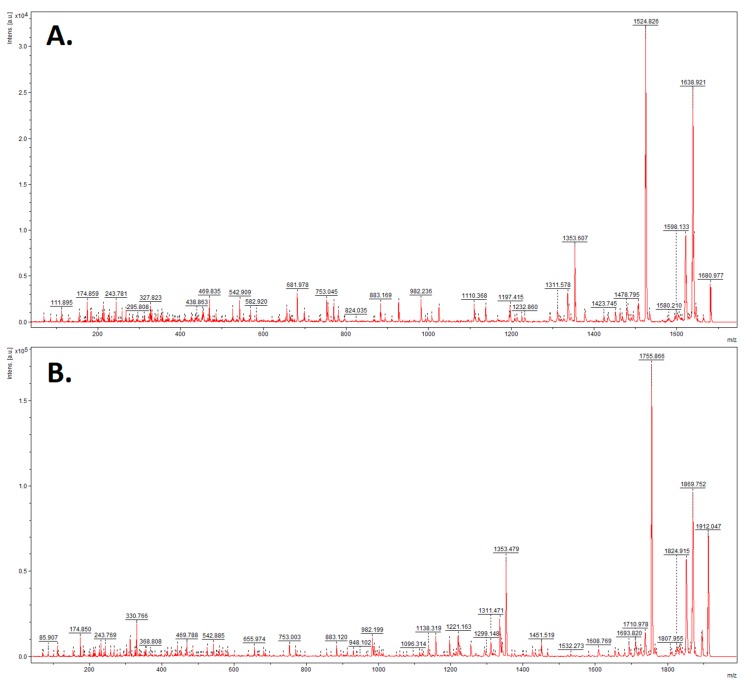
Fragmentation spectra of precursor ions of (**A**) *m*/*z* = 1682.34; (**B**) *m*/*z* = 1913.54 identified as uromodulin.

When comparing the results of the women after GTN treatment with those of the non-pregnant controls, the average value of cross validation was calculated as 56.7% and that of the recognition capability was calculated as 97.1% (Table 2). Peaks of *m*/*z* 1014.98, 1037.47, 1149.51, 1521.33, 1682.34, 1813.54, 1895.43, 2645.54, 2827.52, 3252.07, 3268.81, 3297.96, 3306.06, 7519.26, and 8809.81 were classified as distinguishing between the two groups in the model using GA. By searching the peptide sequence database, uromodulin (two peptides: *m*/*z* = 1682.34 and 1913.54, respectively) and complement C4A (*m*/*z* = 1895.43), which were revealed in the cured patients, were found to be the factors that discriminated between these two groups. Fragmentation of the ion of *m*/*z* 1682.34 resulted in the peptide sequence S.VIDQSRVLNLGPITR.K and fragmentation of signal 1913.54 resulted in the peptide sequence R.SGSVIDQSRVLNLGPITR.K. The fragmentation spectra of *m*/*z* 1682.34 and 1913.54 are presented in Figure 5.

## 4. Discussion

Human chorionic gonadotropin β (βhCG) is currently used as a protein biomarker for GTD. βhCG monitoring is the main method of assessing the process of transformation from benign into malignant forms of the disease [12]. There is no diagnostic tool available to predict that transformation. An increased level of hCG after evacuation of the hydatidiform mole (HM) indicates that there is underlying GTN, yet before there is any hCG increase, we are unable to determine which women will require systemic chemotherapy. Clinical proteomics is a systems approach that facilitates the discovery of novel markers [2]. One of the first reports on proteomics in oncology demonstrated the possibility of using two-dimensional gel electrophoresis (2-DE) to analyze protein-bound fucose in cancer sera [13]. Many researchers pursued the idea of proteomics as a diagnostic tool in different fields of medicine, including gynecological oncology, and focused on this methodology in their research projects [5,6,7,8,9,10,11]. Proteomics has been also applied in the study of GTD by protein profiling of complete moles and normal placenta using surface enhanced laser desorption ionization time-of-flight mass spectrometry (SELDI-ToF MS) with ProteinChip arrays [9]. That study reported a novel method for biomarker discovery that involved laser capture microdissection (LCM), which enabled the evaluation of trophoblast cells and the identification of differences in the protein expression between normal placenta and complete moles. The same study indicated three polypeptides that were specific for the affected tissue in contrast with normal placenta tissue. Another study concentrated on the analysis of protein profiles in tissue from benign and malignant forms of GTD [10]. In that study, using proteomics enabled identification of 17 proteins with altered expression. It was implied that 11 of them (including septin 1, choriomammotropin, cytokeratin 8 and, peroxiredoxin-2) were potential biomarkers of the malignant transformation. In 2011, another study [11] aimed to identify prognostic biomarkers that indicate the malignant transformation of hydatidiform moles. The authors compared the protein profiles of complete benign moles with those of malignant-transformed moles. 18 proteins were found to have been altered in the malignant-transformed group. Among them, chloride intracellular channel protein 1 (CLIC1) was selected by the authors of the 2011 study for further investigation, with results indicating that the levels of CLIC1 expression in choriocarcinoma tissue were higher than in complete hydatidiform mole tissue. Thus, the research showed CLIC1 to be a potential new prognostic biomarker that may indicate patients with hydatidiform moles that are at risk of malignant transformation.

Protein-peptide profiling of urine samples was proposed in our study as a potential way to improve the management of patients with GTN. Urinary proteomics has great potential for use in the discovery of biomarkers of various diseases including neoplastic processes, such as prostate, lung, and thyroid cancers [14,15,16]. Urine contains enough proteins and peptides for analysis and thus is one of the most attractive bio fluids in clinical proteomics [17].

We have demonstrated that the peptide composition of the urine in GTN varies between the groups we evaluated. Our aim for the composition of the control group was to make it as similar as possible to the study group in terms of the clinical data and health conditions of the subjects. Therefore, we matched women diagnosed with GTN prior to treatment with pregnant women as controls because both groups have elevated levels of chorionic gonadotropin (hCG). We wanted to compare the protein profiles of pregnant women with those of patients diagnosed with GTN because we had assumed that in both groups the levels might be similar due to the influence of high levels of hCG during early pregnancy. However, our research revealed the contrary; that the profiles do differ. We also know that, after treatment, hCG should be negative. Therefore, we assumed that pregnant women with increased hCG levels will not constitute a good control group in terms of their protein profiles. For this reason, we enrolled non-pregnant women as controls for our comparison with the post-treatment subjects.

Based on the knowledge that various combinations of biomarkers can increase the sensitivity and specificity of detection [16,18], we used different algorithms to generate combinations of peptide peaks. Our three classification models were genetic algorithm (GA), supervised neural network (SNN), and quick classifier (QC). The best differentiating capacity and most satisfactory values of sensitivity and specificity were associated with the GA model. GA is inspired by the process of natural selection. It chooses the most fitting individuals for subsequent reproduction that will lead to the best next generation of individuals.

When comparing samples from the women diagnosed with GTN prior to treatment with those of the pregnant controls, the nanoLC-MALDI-TOF/TOF-MS/MS technique resulted in the identification of two potential GTN urine biomarkers. We identified two proteins in the patients prior to treatment: Hemoglobin subunit α and complement C4A. In comparing samples from the women post-GTN treatment and those of the non-pregnant control group, uromodulin and complement C4A were found to be the discriminatory factors, with our results revealing that their levels were elevated in the cured group.

Complement is a part of the first line of defense against foreign antigens and unwanted host elements. It is involved in the humoral and adaptive immune response, embryogenesis, and organ repair and development [19]. Complement helps to maintain body homeostasis by regulating immunological and inflammatory processes [20]. The formation of the placenta requires changes in the regulation of the maternal immune system [21,22]. Fetal cells cannot trigger an overactive maternal immune response, including the intense stimulation of the C system [22]. Therefore, an adequate regulation of the C system is a prerequisite for a physiological placentation [23,24]. Mutations in the genes involved in the C system have been detected in patients with recurrent pregnancy loss [25]. Complement C4A/B, a hydrolytic fragment of complement C4, contributes to the propagation of activation pathways involved in the elimination of antigens [26]. The role of complement activation in other tumors has been studied [27], particularly in colorectal cancer [28], lung cancer [29], and breast cancer [30]. The release of complement components has been found to be stimulated as a result of constant exposure to cancer cells’ antigens that mount immune responses. In our study, the higher levels of C4A that we detected in the GTN urine samples suggest that the complement fragments can potentially serve as an indicator of immune stimulation during the course of GTN, and therefore could be considered as an additional diagnostic biomarker.

Uromodulin (UMOD) is an 85 kilo Dalton glycoprotein. It is the most common glycoprotein in urine in physiological conditions, with the only production occurring in the kidneys. Its biological functions remain unclear [31]. Another study showed that protein isolated from pregnancy urine inhibited the antigen-induced proliferation of human lymphocytes in vitro [32]. UMOD might regulate salt transport and play a protective role in urinary tract infection, the formation of kidney stones, kidney injuries, and innate immunity [33]. Elevated UMOD levels may be associated with chronic kidney disease [34]. Therefore, UMOD analysis may be useful in the prediction of renal injury during cancer chemotherapy.

Although hemoglobin (Hb) observed in urine has been poorly investigated, its serum levels have important clinical roles in various diseases [35,36]. Hb concentration in serum has been revealed to be an important predictive factor for the outcome of chemotherapy in the treatment of breast cancer [35]. The authors of that study noticed that low Hb levels may have a negative influence on the response rate of treatment administered to breast cancer patients. Hemoglobin subunit α and β (Hb-α and Hb-β) have also already been identified as novel ovarian serum biomarkers using SELDI-TOF-MS protein chips [36]. The investigation revealed that the combined use of CA125 and Hb could improve the sensitivity of detection rates, in comparison with using CA125 or Hb alone, in the serum of ovarian cancer. Although the meaning of the increased expression of Hb in the urine remains unclear, the combined use of the biomarkers βhCG with Hb-α might also have clinical significance for trophoblastic disease.

We have detected a distinctive protein-peptide profile during the GTD process. Our results suggest that the MS could have a clinical utility in GTD. However, the mechanisms underlying the increased expression of the proteins that our study revealed in urine remain unclear. However, we are aware of the limited number of GTD cases in the study and the lack of a validation set. Further research is required and these issues should be a goal of future studies on trophoblastic disease.

## 5. Conclusions

The peptide profiles of women diagnosed with GTN and of healthy women are distinct. Complement C4A and Hb may be considered an additional diagnostic biomarker of GTN. Uromodulin in cured patients may be a specific fingerprint of past chemotherapy. We suggest that complement C4A fragments can potentially serve as an indicator of immune stimulation in the course of GTD.

## Figures and Tables

**Table 1:** Three different algorithms: genetic algorithm (GA), supervised neural network (SNN), and quick classifier (QC) were used to distinguish between the peptide composition of the group of women diagnosed with GTN before the treatment and the pregnant control group.

Model	Cross Validation (%)	Recognition Capability (%)
GA	68.2	94.4
SNN	50	54
QC	61.8	84.4

**Table 2:** Three different algorithms: genetic algorithm (GA), supervised neural network (SNN), and quick classifier (QC) were used to distinguish between the peptide composition of the group of women diagnosed with GTN after GTN treatment and the non-pregnant control group.

Model	Cross Validation (%)	Recognition Capability (%)
GA	56.7	97.1
SNN	50	52.9
QC	33.6	84
